# A Missing Link between Retrotransposons and Retroviruses

**DOI:** 10.1128/mbio.00187-22

**Published:** 2022-03-15

**Authors:** Jianhua Wang, Guan-Zhu Han

**Affiliations:** a Jiangsu Key Laboratory for Microbes and Functional Genomics, College of Life Sciences, Nanjing Normal University, Nanjing, Jiangsu, China; University of Exeter; University of Pittsburgh

**Keywords:** Comparative genomics, evolution, phylogenetic analysis, retrotransposons, retroviruses

## Abstract

The origin and deep evolution of retroviruses remain largely unclear. It has been proposed that retroviruses might have originated from a Ty3/Gypsy retrotransposon, but all known Ty3/Gypsy retrotransposons are only distantly related to retroviruses. Retroviruses and some plant Athila/Tat elements (within Ty3/Gypsy retrotransposons) independently evolved a dual RNase H domain and an *env*/*env*-like gene. Here, we reported the discovery of a novel lineage of retrotransposons, designated Odin retrotransposons, in the genomes of eight sea anemones (order Actinaria) within the Cnidaria phylum. Odin retrotransposons exhibited unique genome features, encoding a dual RNase H domain (like retroviruses) but no *env* gene (like most Ty3/Gypsy retrotransposons). Phylogenetic analyses based on reverse transcriptase showed that Odin retrotransposons formed a sister group to lokiretroviruses, and lokiretroviruses and Odin retrotransposons together were sister to canonical retroviruses. Moreover, phylogenetic analyses based on RNase H and integrase also supported the hypothesis that Odin retrotransposons were sisters to lokiretroviruses. Lokiretroviruses and canonical retroviruses did not form a monophyletic group, indicating that lokiretroviruses and canonical retroviruses might represent two distinct virus families. Taken together, the discovery of Odin retrotransposons narrowed down the evolutionary gaps between retrotransposons and canonical retroviruses and lokiretroviruses.

## INTRODUCTION

Retroviruses (the *Retroviridae* family) infect a wide range of vertebrates, and their replication requires reverse transcription and integration into host genomes (1–3). While retroviruses usually infect somatic cells (4–7), they occasionally infect germline cells and become integrated into the genome and may be vertically inherited, forming so-called endogenous retroviruses (ERVs) (1–3). Canonical exogenous retroviruses have been typically classified into two subfamilies, namely, *Orthoretrovirinae* (including alpharetroviruses, betaretroviruses, gammaretroviruses, deltaretroviruses, epsilonretroviruses, and lentiviruses) and *Spumaretrovirinae* (foamy viruses) (8). Based on their relationships with exogenous retroviruses, ERVs are traditionally classified into Class I (closely related to gammaretroviruses), Class II (closely related to betaretroviruses), and Class III (closely related to foamy viruses) (4, 9). However, the classification system of exogenous and endogenous retroviruses has not been well incorporated (7, 9).

Recently, a putatively new subfamily of retroviruses, designated lokiretroviruses, has been discovered in the genomes of vertebrates, including lampreys, fishes, amphibians, and reptiles (10). Lokiretroviruses display some unique genome features: (i) Like canonical retroviruses, lokiretroviruses encode a dual RNase H (RH) domain. They acquired a new RH domain, and the preexisting RH domain degenerated to a tether domain (10–13). (ii) Lokiretroviruses encode Env proteins that share detectable sequence similarity with fusion glycoproteins of viruses within the Mononegavirales order (nonsegmented negative-sense single-stranded RNA viruses), but not canonical retroviruses (10). Phylogenetic analyses based on reverse transcriptase (RT) proteins suggest that lokiretroviruses are sister to all the sampled canonical retroviruses, and thus lokiretrovirus was tentatively classified as a novel subfamily within the family *Retroviridae* (10). Thereafter, we used retroviruses to refer to canonical retroviruses and lokiretroviruses, unless otherwise specified. The discovery of lokiretroviruses corroborates the complex evolutionary history of retroviruses (10).

Five reverse transcribing viruses, namely, *Retroviridae*, *Metaviridae* (Ty3/Gypsy retrotransposons), *Pseudoviridae* (Ty1/Copia retrotransposons), *Belpaoviridae* (Bel-Pao retrotransposons), and *Caulimoviridae* (plant pararetroviruses), have recently been unified into the viral order *Ortervirales* (14). Members within the *Ortervirales* order are thought to have originated from a common ancestor (14, 15). Phylogenetic analyses based on RT show that retroviruses are more closely related to the Ty3/Gypsy retrotransposons within the *Ortervirales* order (10, 14–17). Thus, it has been hypothesized that retroviruses might have originated from a Ty3/Gypsy retrotransposon through acquiring an *env* gene (18–20). However, all the known Ty3/Gypsy retrotransposons are only distantly related to retroviruses. It remains largely unclear how retroviruses originated (20).

In this study, we performed systematic mining of retrotransposons that are closely related to retroviruses within 3,624 animal genomes. Intriguingly, we discovered a novel retrotransposon lineage closely related to retroviruses in the genomes of eight sea anemones (order Actinaria) in the phylum Cnidaria. The newly discovered retrotransposons exhibited unique genome features. Evolutionary analyses of the newly discovered retrotransposons provided insights into the diversity and deep evolution of LTR retrotransposons closely related to retroviruses.

## RESULTS

### The discovery of Odin retrotransposons.

**FIG 1:**
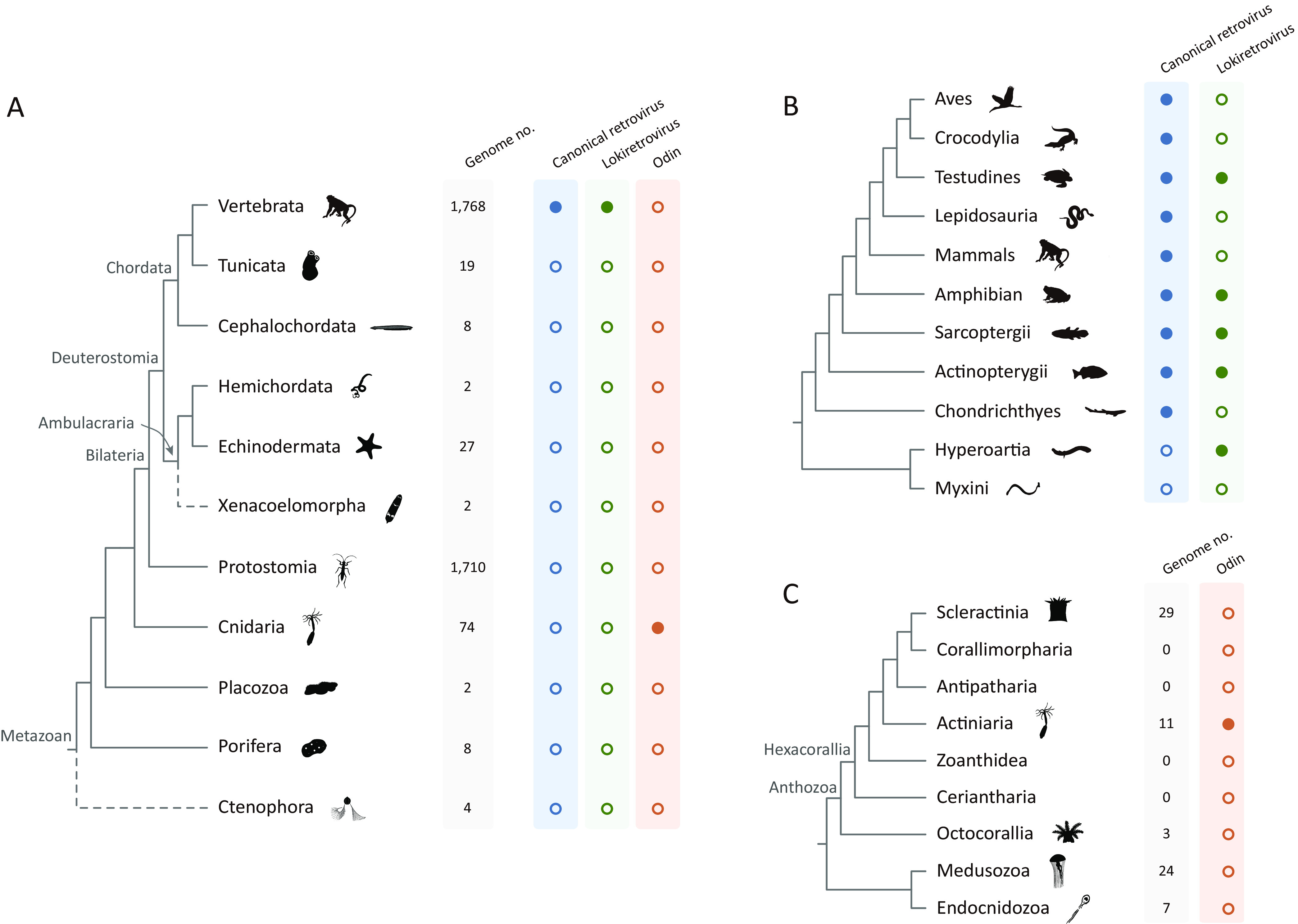
Host distribution of Odin retrotransposons and retroviruses. (A) Distribution of Odin retrotransposons, lokiretroviruses, and canonical retroviruses in metazoans. (B) Distribution of lokiretroviruses and canonical retroviruses in vertebrates. (C) Distribution of Odin retrotransposons in Anthozoa. Phylogenetic relationships of metazoans are based on the literature (34–38). The dashed lines indicate phylogenetic uncertainty. Genome no. represents the number of genomes used in this study. The filled blue, green, and orange circles represent the presence of canonical retroviruses, lokiretroviruses, and Odin retrotransposons in the corresponding animal groups, respectively. The open blue, green, and orange circles represent the absence of canonical retroviruses, lokiretroviruses, and Odin retrotransposons in the corresponding animal groups, respectively.

**FIG 2:**
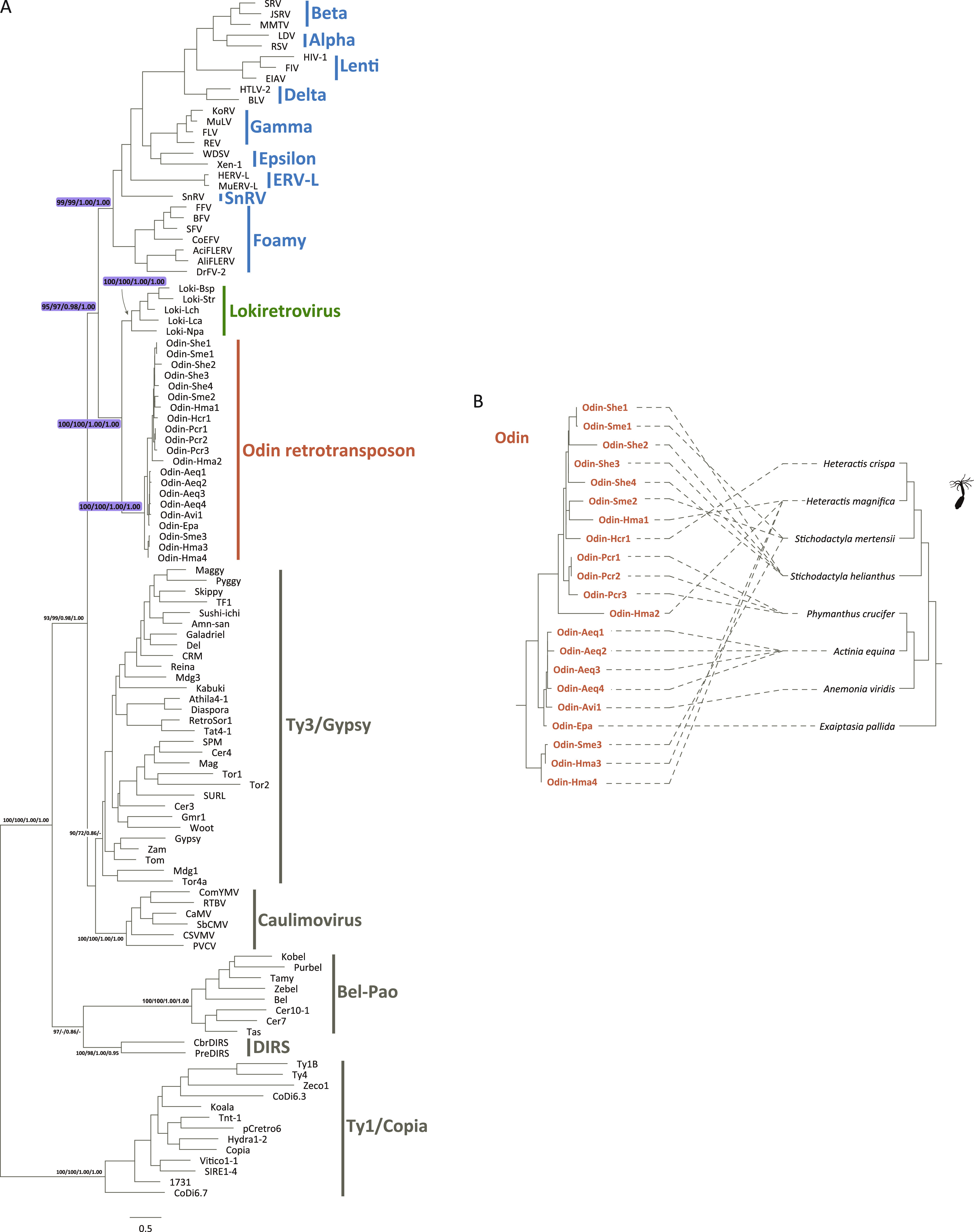
Phylogenetic relationships among Odin retrotransposons, representative canonical retroviruses, lokiretroviruses, and LTR retrotransposons. (A) Phylogenetic trees were reconstructed based on RT proteins of Odin retrotransposons, representative canonical retroviruses, lokiretroviruses, and LTR retrotransposons. (B) Comparison of Odin retrotransposon and host phylogenies. The left is the Odin retrotransposon phylogeny, whereas the right is the host phylogeny based on the literature (39).

To investigate the origin of retroviruses, we used a similarity search and phylogenetic analyses combined approach to screen retroelements that are closely related to retroviruses within 3,624 animal genomes (1,768 Vertebrata, 1,710 Protostomia, 74 Cnidaria, 27 Echinodermata, 19 Tunicata, 8 Cephalochordata, 8 Porifera, 4 Ctenophora, 2 Hemichordata, 2 Xenacoelomorpha, and 2 Placozoa) (Fig. 1A; Table S1 and S2) retrieved from NCBI. Endogenous canonical retroviruses and lokiretroviruses have been only identified within the genomes of vertebrates (Fig. 1B) (7, 10). Intriguingly, we identified a novel lineage of retrotransposons in the genomes of eight sea anemones (order Actinaria) within the Cnidaria phylum (Fig. 1A to C and Fig. 2A). We designated the retrotransposon lineage Odin retrotransposons following the name of Odin in Norse mythology, the blood brother of Loki after whom lokiretroviruses was named (10). The copy numbers of Odin retrotransposons were generally low within cnidaria genomes, ranging from one in Exaiptasia pallida to six in Heteractis magnifica (Table S3). Moreover, several Odin retrotransposons integrated into host genomes in recent time (from 0 to 4.66 million years ago; Table S3), suggesting that some Odin retrotransposons might still be active in their host genomes.

10.1128/mbio.00187-22.2 TABLE S1 Information of queries used in this study. Download Table S1, PDF file, 0.06 MB. Copyright © 2022 Wang and Han. 2022 Wang and Han. https://creativecommons.org/licenses/by/4.0/ This content is distributed under the terms of the Creative Commons Attribution 4.0 International license.

10.1128/mbio.00187-22.3 TABLE S2 Information of sequences used for phylogenetic analyses. Download Table S2, PDF file, 0.09 MB. Copyright © 2022 Wang and Han. 2022 Wang and Han. https://creativecommons.org/licenses/by/4.0/ This content is distributed under the terms of the Creative Commons Attribution 4.0 International license.

10.1128/mbio.00187-22.4 TABLE S3 Information and location of Odin retrotransposons. Download Table S3, PDF file, 0.1 MB. Copyright © 2022 Wang and Han. 2022 Wang and Han. https://creativecommons.org/licenses/by/4.0/ This content is distributed under the terms of the Creative Commons Attribution 4.0 International license.

**FIG 3:**
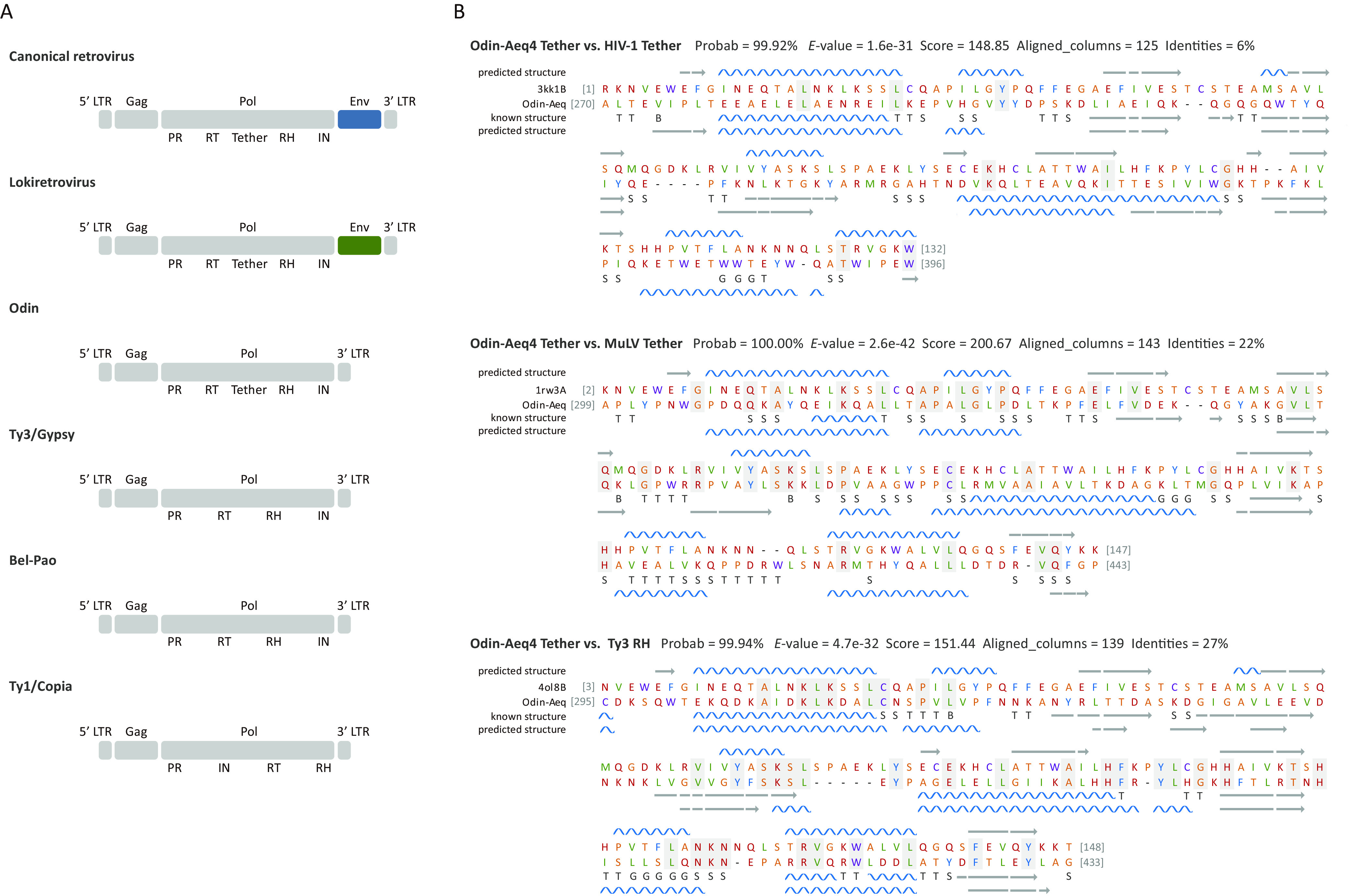
Domain architectures and secondary structure of retroviruses and retrotransposons. (A) Comparison of domain architectures of canonical retroviruses, lokiretroviruses, Odin retrotransposons, and other retrotransposons. (B) Comparison of secondary structure between the tether domain of Odin-Aeq4 and that of HIV-1 (PDB accession no.: 3KK1.B) and MuLV (PDB accession no.: 1RW3.A), and between the tether domain of Odin-Aeq4 and the RH domain of Ty3 (PDB accession no.: 4OL8.A). The blue helices and orange arrows represent α-helices and β-strands, respectively. “T,” “S,” and “G” characters represent the following: T = hydrogen-bonded turn, S = bend, and G = 310 helix.

Retrovirus genomes encode dual RH domains and *env* genes, whereas most long terminal repeat (LTR) retrotransposons do not encode dual RH domains or *env* genes [with a few exceptions, such as some land plant Athila/Tat elements within Ty3/Gypsy retrotransposons; (12, 13)]. Retroviruses and Athila/Tat elements independently evolved a dual RNase H domain and an *env*/*env*-like gene (12, 13). Odin retrotransposons encoded two putative genes that were common to LTR retrotransposons, namely, *gag* and *pol*, flanked by two LTRs (Fig. 3A). No *env*-like gene was predicted within Odin retrotransposons. Odin retrotransposon Pol proteins comprised four domains, including protease (PR), RT, RH, and integrase (IN) (Fig. 3A). Interestingly, like retroviruses and unlike most LTR retrotransposons, Odin retrotransposons encoded a dual RH domain that consisted of a tether domain derived from the degenerated Ty3/Gypsy retrotransposon RH domain and a newly acquired RH domain (10–13). The detectable structural similarity was found between the tether domains of Odin retrotransposon and canonical retroviruses (for example, Odin retrotransposon versus human immunodeficiency virus type 1 [HIV-1]: probability = 99.92%, *E* value = 1.6 × 10^−31^, identities = 6%; Odin retrotransposon versus murine leukemia virus [MuLV]: probability = 100%, *E* value = 2.6 × 10^−42^, identities = 22%) (Fig. 3B). Moreover, the tether domain of Odin retrotransposon shared detectable structural similarity with the RH domain of the Ty3 retrotransposon (probability = 99.94%, *E* value = 4.7 × 10^−32^, identities = 27%) (Fig. 3B). Taken together, our results showed that Odin retrotransposons exhibited unique genome features, encoding a dual RNase H domain (like retroviruses) but no *env* gene (like most of Ty3/Gypsy retrotransposons).

### Odin retrotransposons are sister to lokiretroviruses.

To explore the evolutionary relationship among Odin retrotransposons, retroviruses, and other *Ortervirales* members, we performed phylogenetic analyses based on RT protein alignments generated by two different methods (MAFFT with the L-INS-I strategy [align-Ma] and PROMAL3D with the default parameters [align-3D]) using two tree reconstruction algorithms (maximum likelihood and Bayesian inference) (Table S2) and obtained four largely consistent phylogenies. Phylogenetic analyses showed that Odin retrotransposons formed a sister group of lokiretroviruses with robust support values (ultrafast bootstrap approximation [UFBoot] = 100% for both alignments and Bayesian posterior probability [BPP] = 1.00 for both alignments) (Fig. 2A). Canonical retroviruses (*Orthoretrovirinae* and *Spumaretrovirinae*) did not form a monophyletic group with lokiretroviruses. Rather, Odin retrotransposons and lokiretroviruses formed a sister group to canonical retroviruses (UFBoot = 95% and 97% for align-Ma and align-3D, respectively; BPP = 0.98 and 1.00 for align-Ma and align-3D, respectively) (Fig. 2A). Taken together, our results suggested that Odin retrotransposons were sister to lokiretroviruses and the closest known retrotransposon relatives of retroviruses (canonical retroviruses and lokiretroviruses).

### Frequent host switching of Odin retrotransposons.

Initially, we found that the phylogeny of Odin retrotransposons based on RT protein sequences was generally inconsistent with host phylogeny (Fig. 2B). We further used an event-based approach to compare the congruence of phylogenies between Odin retrotransposons and their hosts at the species level and the family level. We found significant incongruence between phylogenies of Odin retrotransposons and their hosts at the species level (*P* > 0.05; Table 1) and at the family level (*P* > 0.05; Table 1). These results suggested that Odin retrotransposons mainly evolved through frequent host switching (horizontal transfers).

**TABLE 1:** Test for congruence of phylogenies between Odin retrotransposons and their hosts

Datasets	Event costs^*a*^	Total cost	No. of events					*P* value^*b*^
			Cospeciation	Duplication	Duplication and host switching	Loss	Failure to diverge	
Species	0, 1, 2, 1, 1	27	3	8	9	1	0	*P* > 0.05
Species	0, 1, 1, 2, 0	18	2	6	12	0	0	*P* > 0.05
Species	−1, 0, 0, 0, 0	−5	5	6	9	9	0	*P* > 0.05
Family	0, 1, 2, 1, 1	21	2	15	3	0	0	*P* > 0.05
Family	0, 1, 1, 2, 0	18	2	15	3	0	0	*P* > 0.05
Family	−1, 0, 0, 0, 0	−2	2	15	3	0	0	*P* > 0.05

a Event cost schemes are for cospeciation, duplication, duplication with host switch, loss, and failure to diverge, respectively.

b *P* value represents statistical analysis results by using the method of random parasite tree with a sample size of 500.

### The evolutionary history of lokiretrovirus RH and IN domains.

**FIG 4:**
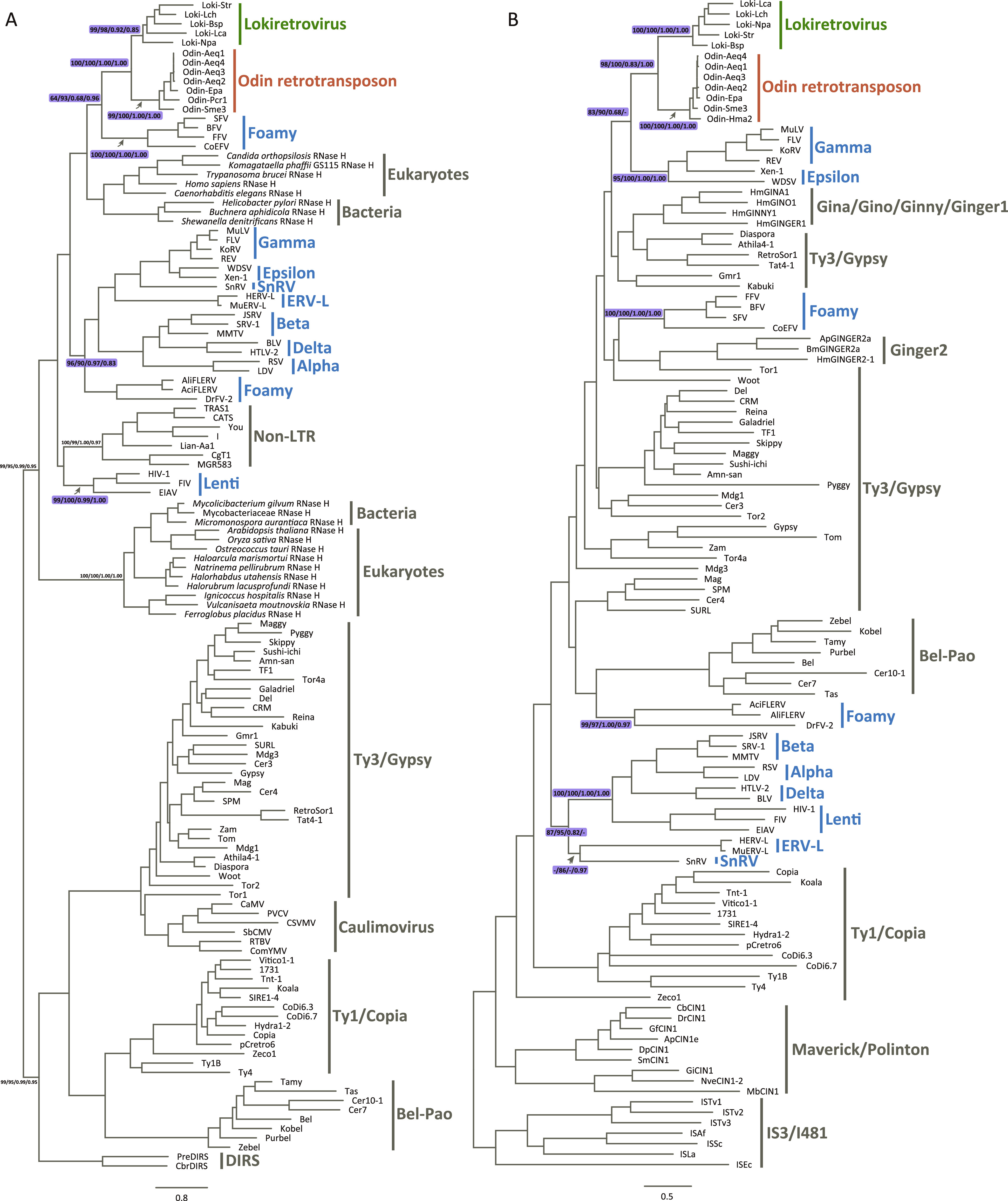
Phylogenetic trees of RH and IN domains. (A) Phylogenetic tree of RH domains. (B) Phylogenetic tree of IN domains. The support values are listed in the order of UFBoot for align-Ma/UFBoot for align-3D/BPP for align-Ma/BPP for align-3D.

To further explore the evolutionary history of Odin retrotransposons and retroviruses, we performed phylogenetic analyses based on their RH and IN domains. Broadly consistent with previous studies (10, 13), both RH and IN domains of canonical retroviruses clustered into multiple groups, suggesting that canonical retroviruses frequently replaced their RH and IN domains during their evolutionary history (Fig. 4) (10). Consistent with phylogenetic analyses of the RT domain, we found that Odin retrotransposons were sister to lokiretroviruses for both RH and IN domains with robust support values (RH domain: UFBoot = 100% for both alignments, BPP = 1.00 for both alignments; IN domain: UFBoot = 98% and 100% for align-Ma and align-3D, respectively, and BPP = 0.83 and 1.00 for align-Ma and align-3D, respectively) (Fig. 4). It should be noted that, because phylogenetic analyses of RH and IN are notoriously problematic (10), we could not infer a robust evolutionary relationship among Odin retrotransposons, lokiretroviruses, and canonical retroviruses for RH or IN domains. Nevertheless, our phylogenetic analyses of RH and IN domains further supported that Odin retrotransposons were sister to lokiretroviruses.

## DISCUSSION

In this study, we discovered a novel lineage of retrotransposons, referred to as Odin retrotransposons, within eight sea anemones (order Actiniaria, phylum Cnidaria). Odin retrotransposons exhibited unique genome features, encoding a dual RNase H domain (like retroviruses) but no Envelope protein (like most of Ty3/Gypsy retrotransposons). Our phylogenetic analyses showed that Odin retrotransposons formed a sister group to lokiretroviruses, and Odin retrotransposons and lokiretroviruses were sisters to canonical retroviruses. Lokiretroviruses and canonical retroviruses did not form a monophyletic group. Therefore, Odin retrotransposons were the closest known retrotransposon relatives to retroviruses (canonical retroviruses and lokiretroviruses). Retroviruses have long been thought to have originated from an ancient Ty3/Gypsy retrotransposon (15, 16, 20), but the sampled Ty3/Gypsy retrotransposons were only distantly related to retroviruses. The discovery of Odin retrotransposons narrowed down the evolutionary gap between Ty3/Gypsy retrotransposons and retroviruses. Odin retrotransposons might represent the modern descendants of those long-sought-after Ty3/Gypsy retrotransposons.

Ty3/Gypsy retrotransposons typically encode two common genes (*gag* and *pol*), whereas both canonical retroviruses and lokiretroviruses encode an additional gene, *env*, besides *gag* and *pol* genes (18). Moreover, most of Ty3/Gypsy retrotransposon (with land plant Athila/Tat retrotransposons as the exception) Pol proteins comprise PR, RT, RH, and IN domains, whereas both canonical retroviruses and lokiretroviruses encode the dual RH domain. The preexisting RH domain degenerated to a tether domain and a new RH domain was acquired (10–13). Interestingly, Odin retrotransposons also possess the dual RH domain with a degraded RH domain (the tether domain) but no Env proteins. Therefore, the genome architecture of Odin retrotransposons might represent an intermediate formed between Ty3/Gypsy retrotransposons and canonical retroviruses/lokiretroviruses.

The sequence identity between the tether domains of canonical retroviruses and the RH domains was too low to be used for phylogenetic analyses, but the tether domains of Odin retrotransposons and lokiretroviruses shared detectable structural similarity with the tether domains of canonical retroviruses (HIV-1 and MuLV) and the RH domain of Ty3/Gypsy retrotransposons (the Ty3 retrotransposon) (10). Moreover, phylogenetic analyses of RT proteins showed that Odin retrotransposons, lokiretroviruses, and canonical retroviruses clustered together. Therefore, we inferred that the degradation of the preexisting RH domain occurred in the most recent common ancestor (MRCA) of Odin retrotransposons and retroviruses. For canonical retroviruses, the newly acquired RH domains did not cluster together, indicating canonical retroviruses replaced their RH domains multiple times (10, 13).

**FIG 5:**
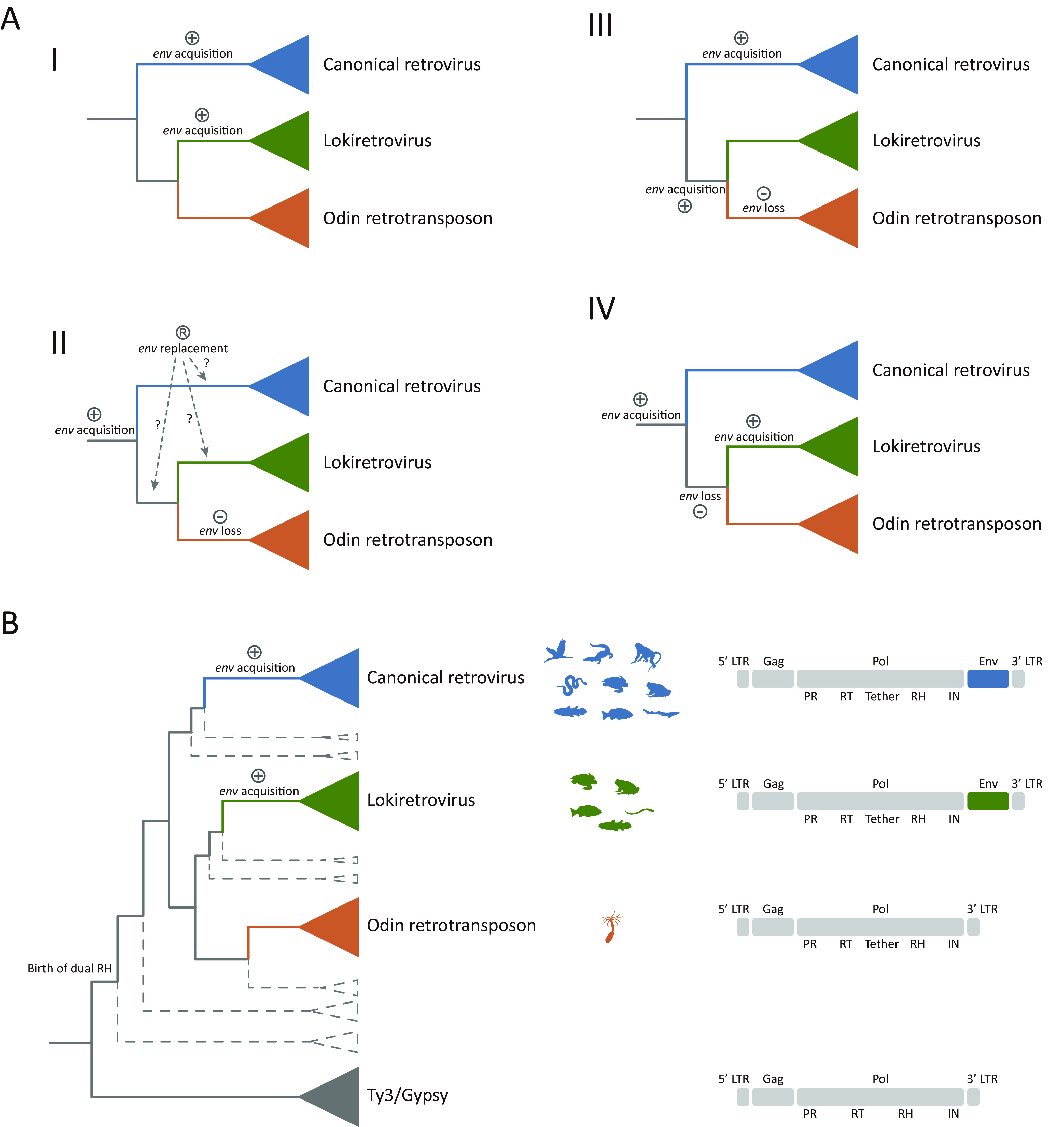
Evolutionary scenarios for the origin of retroviruses. (A) Four possible scenarios of the origin of retroviruses. Scenario I: Canonical retroviruses and lokiretroviruses acquired Env proteins independently from different sources. Scenario II: The MRCA of Odin retrotransposons and retroviruses acquired an *env* gene. Canonical retroviruses, lokiretroviruses, or the MRCA of lokiretroviruses and Odin retrotransposons replaced their *env* genes during the evolutionary course. Odin retrotransposons then lost the *env* gene. Scenario III: Canonical retroviruses and the MRCA of lokiretroviruses and Odin retrotransposons acquired *env* genes independently and then Odin retrotransposons lost the *env* gene. Scenario IV: The MRCA of Odin retrotransposons and retroviruses acquired an *env* gene, and the MRCA of Odin retrotransposons and lokiretroviruses lost the *env* gene. Lokiretroviruses then acquired a new *env* gene. (B) Model for the retrovirus origin. Canonical retroviruses and lokiretroviruses originated independently through the acquisitions of Env proteins from different sources. Dashed lines represent Odin-like retrotransposons that remain to be identified.

Our phylogenetic analyses provided a crucial framework for investigating the origin and evolution of retroviruses (canonical retroviruses and lokiretroviruses). The MRCA of Odin retrotransposons and retroviruses was likely to be an LTR retrotransposon with a dual RH domain. Env proteins of canonical retroviruses and lokiretroviruses did not share detectable similarities, but instead, lokiretrovirus Env proteins shared detectable similarities with fusion glycoproteins of viruses within the Mononegavirales, suggesting that Env proteins of canonical retroviruses and lokiretroviruses were likely to be of different origins (10). Based on currently available information, at least four different evolutionary scenarios could be conceived to account for the origin of retroviruses. Independent origins of canonical retroviruses and lokiretroviruses (Fig. 5A and I). Canonical retroviruses and lokiretroviruses originated independently by acquiring Env proteins from different sources. The ancestor of lokiretroviruses might have acquired an Env protein from negative-sense single-stranded viruses (10). Odin retrotransposons might represent one of the modern descendants of these retrotransposon ancestors of retroviruses. The MRCA of Odin retrotransposons and retroviruses acquired an *env* gene. The *env* gene was replaced along with the evolution of canonical retroviruses, lokiretroviruses, or the MRCA of lokiretroviruses and Odin retrotransposons. The *env* gene was then lost in Odin retrotransposons (Fig. 5A, II). Canonical retroviruses and the MRCA of lokiretroviruses and Odin retrotransposons independently acquired *env* genes, and the *env* gene was lost during the evolutionary course of Odin retrotransposons (Fig. 5A, III). The MRCA of Odin retrotransposons and retroviruses acquired an *env* gene. The *env* gene was lost in the MRCA of Odin retrotransposons and lokiretroviruses. But lokiretroviruses acquired a new *env* gene during their evolutionary course (Fig. 5A, IV). Among these evolutionary scenarios, scenario I (two gain events) was more parsimonious than the other scenarios (at least three steps). Therefore, we prefer the hypothesis that canonical retroviruses and lokiretroviruses originated independently by acquiring Env proteins from different sources (Fig. 5B). If so, more Odin-like retrotransposons with a dual RH domain but without Env proteins (indicated by dashed lines in Fig. 5B) await to be discovered possibly within the genomes from metazoan groups that are underrepresented in genome sequencing projects (outside Vertebrata and Protostomia). Moreover, given the small sample of Odin retrotransposons currently identified, a larger sample is required to further corroborate the lack of an *env* gene in the group.

Both canonical retroviruses and lokiretroviruses have been thought to infect vertebrates exclusively and widely. Odin retrotransposons were discovered in only eight sea anemones (only 11 anemone genomes were screened) with low copy numbers [ranging from one in Exaiptasia pallida to six in Heteractis magnifica (Table S3)]. Compared with Odin retrotransposons, canonical retroviruses and lokiretroviruses appear to be more widespread. The potential for infectivity (i.e., the presence of an *env* gene) of retroviruses and lokiretrovirus might entail the likelihood of host range expansion. However, evolutionary gaps remain between Odin retrotransposons of cnidaria and canonical retroviruses/lokiretroviruses of vertebrates. Two possibilities might account for the gaps: (i) horizontal transfer of Odin-like retrotransposons occurred between cnidaria and vertebrates; (ii) compared with many Vertebrata genomes (1,768) and Protostomia genomes (1,710), only 146 genomes from metazoans outside vertebrates and protostomia have been sequenced, and many more Odin or Odin-like retrotransposons (illustrated using dashed lines in Fig. 5B) might exist within metazoans.

In our previous study (10), lokiretroviruses were classified as a subfamily of retroviruses. However, our phylogenetic analyses show that lokiretroviruses and canonical retroviruses do not form a monophyletic group after adding the newly discovered Odin retrotransposons. Moreover, canonical retroviruses and lokiretroviruses might have acquired their Env proteins independently. Therefore, lokiretrovirus might represent a misnomer, and we think it is necessary to rename lokiretrovirus to lokiortervirus, which reflects that it belongs to the viral order *Ortervirales*. Unlike most Ty3/Gypsy retrotransposons (*Metaviridae*), Odin retrotransposons encode the dual RH domain, and Odin retrotransposons do not cluster within the diversity of Ty3/Gypsy retrotransposons. Taken together, we propose that lokiorterviruses and Odin retrotransposons can be tentatively classified into two novel viral families (*Lokiorterviridae* and *Odinorterviridae*) within the order *Ortervirales*.

## MATERIALS AND METHODS

### The discovery of Odin retrotransposons.

We used a similarity search and phylogenetic analyses combined approach (10) to mine retroelements that are closely related to retroviruses. First, we used the tBLASTn algorithm to screen homologs of retrovirus RT proteins within 3,624 animal genomes available in NCBI (including 1,768 Vertebrata, 1,710 Protostomia, 74 Cnidaria, 27 Echinodermata, 19 Tunicata, 8 Cephalochordata, 8 Porifera, 4 Ctenophora, 2 Hemichordata, 2 Xenacoelomorpha, and 2 Placozoa) using 10 representative canonical retrovirus and lokiretrovirus RT proteins as queries with an *E* cutoff value of 10^−5^ (Fig. 1; Table S1). We then performed phylogenetic analyses of significant hits with the length of >50 amino acids (aa) and RT proteins of representative canonical retroviruses, lokiretroviruses, and retrotransposons (21). RT protein sequences were aligned using MAFFT v7.475 (22). The initial large-scale phylogenetic analyses were performed using an approximate maximum likelihood method implemented in FastTree 2.1.10 (23). Some significant hits from several sea anemones, referred to as Odin retrotransposons form a sister group to lokiretroviruses. To further confirm the distribution of Odin retrotransposons, we performed a second round of similarity search with an Odin retrotransposon RT protein of Actinia equine (NCBI accession no. WHPX01000927.1, from 148,958 to 149,728; referred to as Odin-Aeq4) as the query and an *E* cutoff value of 10^−5^. Phylogenetic analyses were performed using the approaches described above. Significant hits that cluster with Odin retrotransposons were retrieved for further study.

### Genome structure reconstruction and secondary structure prediction.

We bidirectionally extended the retrieved Odin retrotransposon significant hits and predicted the domain architecture using the conserved domain (CD) search with default parameters (24). LTR_Finder was used to predict the 5′- and 3′-LTRs with default parameters (25). The Phyre2 web was used to compare the secondary structure between the tether domain of an Odin retrotransposon within Actinia equine (namely, Odin-Aeq4), the tether domains of HIV-1 and MuLV, and between the tether domain of Odin-Aeq4 and the RH domain of the Ty3 retrotransposon (26).

### Phylogenetic analyses.

To explore the relationship among Odin retrotransposons, canonical retroviruses, lokiretroviruses, and LTR retrotransposons, their RT, RH, and IN protein sequences were used to perform phylogenetic analyses (Table S2 and S3). Sequences were aligned using MAFFT v7.475 with the L-INS-I strategy and PROMALS3D, an alignment tool based on enhanced information from database searches, secondary structure prediction, and 3D structures, with the default parameters, and ambiguous regions were manually removed (Data Set S1 contains original alignments, and Data sets S2-S7 are alignments manually refined) (22, 27). The length of the RT alignments is 252 aa and 326 aa for align-Ma and align-3D, respectively (Data Set S2 and S3). The length of the RH alignments is 198 aa and 191 aa for align-Ma and align-3D, respectively (Data Set S4 and S5). The length of the IN alignments is 270 aa and 225 aa for align-Ma and align-3D, respectively (Data Set S6 and S7). A maximum likelihood approach implemented in IQ-Tree 2 was used to perform phylogenetic analyses (28). The best-fit models were estimated by Model Finder (29). The best-fit model for each alignment is as follows: LG+R6 for multiple sequence alignments of RT and RH proteins generated by MAFFT, LG+R5 for multiple sequence alignments of RT and RH proteins generated by PROMALS3D, and LG+F+R6 for both multiple sequence alignments of IN proteins. Node supports were assessed using ultrafast bootstrap approximation with 1,000 replicates (30). Moreover, we also performed phylogenetic analyses using a Bayesian method implemented in MrBayes 3.2.7a (31). The best-fit models were selected using ModelTest-NG (32). The best-fit model for each alignment is as follows: LG+G4 for all multiple alignments of RT and RH proteins and LG+I+G4 for all multiple alignments of IN proteins.

10.1128/mbio.00187-22.5 DATA SET S1 Original alignments of RT proteins, RH proteins, and IN proteins by different methods. Download Data Set S1, TXT file, 0.2 MB. Copyright © 2022 Wang and Han. 2022 Wang and Han. https://creativecommons.org/licenses/by/4.0/ This content is distributed under the terms of the Creative Commons Attribution 4.0 International license.

10.1128/mbio.00187-22.6 DATA SET S2 Multiple sequence alignment of RT proteins of representative retroviruses, Odin retrotransposons, and other LTR retrotransposons generated by MAFFT and used for phylogenetic analyses. Download Data Set S2, TXT file, 0.03 MB. Copyright © 2022 Wang and Han. 2022 Wang and Han. https://creativecommons.org/licenses/by/4.0/ This content is distributed under the terms of the Creative Commons Attribution 4.0 International license.

10.1128/mbio.00187-22.7 DATA SET S3 Multiple sequence alignment of RT proteins of representative retroviruses, Odin retrotransposons, and other LTR retrotransposons generated by PROMALS3D and used for phylogenetic analyses. Download Data Set S3, TXT file, 0.04 MB. Copyright © 2022 Wang and Han. 2022 Wang and Han. https://creativecommons.org/licenses/by/4.0/ This content is distributed under the terms of the Creative Commons Attribution 4.0 International license.

10.1128/mbio.00187-22.8 DATA SET S4 Multiple sequence alignment of RH proteins of representative retroviruses, Odin retrotransposons, other LTR retrotransposons, and host RH proteins generated by MAFFT and used for phylogenetic analyses. Download Data Set S4, TXT file, 0.02 MB. Copyright © 2022 Wang and Han. 2022 Wang and Han. https://creativecommons.org/licenses/by/4.0/ This content is distributed under the terms of the Creative Commons Attribution 4.0 International license.

10.1128/mbio.00187-22.9 DATA SET S5 Multiple sequence alignment of RH proteins of representative retroviruses, Odin retrotransposons, other LTR retrotransposons, and host RH proteins generated by PROMALS3D and used for phylogenetic analyses. Download Data Set S5, TXT file, 0.02 MB. Copyright © 2022 Wang and Han. 2022 Wang and Han. https://creativecommons.org/licenses/by/4.0/ This content is distributed under the terms of the Creative Commons Attribution 4.0 International license.

10.1128/mbio.00187-22.10 DATA SET S6 Multiple sequence alignment of IN proteins of representative retroviruses, Odin retrotransposons, other LTR retrotransposons, and DNA transposons generated by MAFFT and used for phylogenetic analyses. Download Data Set S6, TXT file, 0.03 MB. Copyright © 2022 Wang and Han. 2022 Wang and Han. https://creativecommons.org/licenses/by/4.0/ This content is distributed under the terms of the Creative Commons Attribution 4.0 International license.

10.1128/mbio.00187-22.11 DATA SET S7 Multiple sequence alignment of IN proteins of representative retroviruses, Odin retrotransposons, other LTR retrotransposons, and DNA transposons generated by PROMALS3D and used for phylogenetic analyses. Download Data Set S7, TXT file, 0.02 MB. Copyright © 2022 Wang and Han. 2022 Wang and Han. https://creativecommons.org/licenses/by/4.0/ This content is distributed under the terms of the Creative Commons Attribution 4.0 International license.

### Phylogeny congruence test.

Jane 4 was used to compare the congruence between the phylogenies of Odin retrotransposons and their hosts (33). Three sets of cost values for cospeciation, duplication, duplication with host switch, loss, and failure to diverge were used, including 0, 1, 2, 1, 1; -1, 0, 0, 0, 0; and 0, 1, 1, 2, 0 (10). *P* values were estimated using the random parasite tree method with a sample size of 500.
