# Supplementary material for: A Missing Link between Retrotransposons and Retroviruses
Source: mBio. 2022 Mar 15;13(2):e00187-22. doi: 10.1128/mbio.00187-22 (PMC9040795; doi:10.1128/mbio.00187-22)
Supplement: TABLE S1 [file mbio.00187-22-st001.pdf]

Table S1. Information of queries used in this study

Name	Abbreviation	Accession No.
Friend murine leukemia virus	MLV	NP_040333.1
Walleye dermal sarcoma virus	WDSV	NP_045937.2
Mouse mammary tumor virus	MMTV	NP_056880.1
Human endogenous retroviruses type L	HERV-L	Ref. 1
Eastern chimpanzee simian foamy virus	SFVcpz	YP_009508551.1
Lokiretrovirus	Loki-Str	Ref. 2
Avian leukemia virus	ALV	YP_004222728.1
Human T-lymphotropic virus 2	HTLV-2	NP_041003.3
Human immunodeficiency virus 1	HIV-1	NP_057849.4
Snakehead retrovirus	SnRV	NP_043924.1

References

1. Vargiu L, Rodriguez-Tome P, Sperber GO, Cadeddu M, Grandi N, Blikstad V, Tramontano E, Blomberg J. 2016. Classification and characterization of human endogenous retroviruses; mosaic forms are common. Retrovirology 13:7.
2. Wang J, Han GZ. 2021. A Sister Lineage of Sampled Retroviruses Corroborates the Complex Evolution of Retroviruses. Mol Biol Evol 38:1031-1039.
