# Supplementary material for: A Missing Link between Retrotransposons and Retroviruses
Source: mBio. 2022 Mar 15;13(2):e00187-22. doi: 10.1128/mbio.00187-22 (PMC9040795; doi:10.1128/mbio.00187-22)
Supplement: TABLE S2 [file mbio.00187-22-st002.pdf]

Table S2. Information of sequences used for phylogenetic analyses

Type/Group	Name	Accession No.
Bacterium	*Mycolicibacterium gilvum* RNase H	WP_011893771.1
Bacterium	Mycobacteriaceae RNase H	WP_011560725.1
Bacterium	*Micromonospora aurantiaca* RNase H	WP_013287786.1
Bacterium	*Buchnera aphidicola* RNase H	WP_011091358.1
Bacterium	*Shewanella denitrificans* RNase H	WP_011496458.1
Bacterium	*Helicobacter pylori* RNase H	WP_001155465.1
Belpaoviridae	Kobel	Gypsy Database 2.0
Belpaoviridae	Tamy	Gypsy Database 2.0
Belpaoviridae	Zebel	Gypsy Database 2.0
Belpaoviridae	Bel	Gypsy Database 2.0
Belpaoviridae	Purbel	Gypsy Database 2.0
Belpaoviridae	Cer10-1	Gypsy Database 2.0
Belpaoviridae	ApBIN1h	Gypsy Database 2.0
Belpaoviridae	ApBIN1j	Gypsy Database 2.0
Caulimovirus	CaMV	Gypsy Database 2.0
Caulimovirus	SbCMV	Gypsy Database 2.0
Caulimovirus	ComYMV	Gypsy Database 2.0
Caulimovirus	RTBV	Gypsy Database 2.0
Caulimovirus	CSVMV	Gypsy Database 2.0
Caulimovirus	PVCV	Gypsy Database 2.0
DIRS	*Caenorhabditis briggsae* AF16 DIRS	CAP25970.2
DIRS	*Panagrellus redivivus* DIRS	CAA43185.1
Eukaryote	*Arabidopsis thaliana* RNase H	AAF24611.1
Eukaryote	*Oryza sativa* RNase H	AAK54296.1
Eukaryote	*Ostreococcus tauri* RNase H	XP_003080907.1
Eukaryote	*Ignicoccus hospitalis* RNase H	WP_012123294.1
Eukaryote	*Vulcanisaeta moutnovskia* RNase H	WP_013604220.1
Eukaryote	*Ferroglobus placidus* RNase H	WP_012965086.1
Eukaryote	*Haloarcula marismortui* RNase H	AAV48010.1
Eukaryote	*Natrinema pellirubrum* RNase H	WP_006182114.1
Eukaryote	*Halorhabdus utahensis* RNase H	WP_015788958.1
Eukaryote	*Halorubrum lacusprofundi* RNase H	WP_012659552.1
Eukaryote	*Candida orthopsilosis* RNase H	XP_003871485.1
Eukaryote	*Komagataella phaffii* GS115 RNase H	XP_002490024.1
Eukaryote	*Trypanosoma brucei* RNase H	AAC47537.1
Eukaryote	*Homo sapiens* RNase H	AAV38476.1
Eukaryote	*Caenorhabditis elegans* RNase H	NP_001040786.1
Gina	HmGINA1	Gypsy Database 2.0
Ginger1	HmGINGER1	Gypsy Database 2.0
Ginger2	HmGINGER2-1	Gypsy Database 2.0
Ginger2	ApGINGER2a	Gypsy Database 2.0

Ginger2	BmGINGER2a	Gypsy Database 2.0
Ginny	HmGINNY1	Gypsy Database 2.0
Gino	HmGINO1	Gypsy Database 2.0
IS3/IS481	ISSc	Gypsy Database 2.0
IS3/IS481	ISAf	Gypsy Database 2.0
IS3/IS481	ISLa	Gypsy Database 2.0
IS3/IS481	ISTv1	Gypsy Database 2.0
IS3/IS481	ISEc	Gypsy Database 2.0
IS3/IS481	ISTv3	Gypsy Database 2.0
IS3/IS481	ISTv2	Gypsy Database 2.0
Maverick/Polinton	CbCIN1	Gypsy Database 2.0
Maverick/Polinton	DrCIN1	Gypsy Database 2.0
Maverick/Polinton	GfCIN1	Gypsy Database 2.0
Maverick/Polinton	ApCIN1	Gypsy Database 2.0
Maverick/Polinton	DpCIN1	Gypsy Database 2.0
Maverick/Polinton	SmCIN1	Gypsy Database 2.0
Maverick/Polinton	GiCIN1	Gypsy Database 2.0
Maverick/Polinton	NveCIN1-2	Gypsy Database 2.0
Maverick/Polinton	MbCIN1-2	Gypsy Database 2.0
Metaviridae	Maggy	Gypsy Database 2.0
Metaviridae	Pyggy	Gypsy Database 2.0
Metaviridae	Skippy	Gypsy Database 2.0
Metaviridae	TF1	Gypsy Database 2.0
Metaviridae	Amn-san	Gypsy Database 2.0
Metaviridae	Sushi-ichi	Gypsy Database 2.0
Metaviridae	Del	Gypsy Database 2.0
Metaviridae	Galadriel	Gypsy Database 2.0
Metaviridae	CRM	Gypsy Database 2.0
Metaviridae	Reina	Gypsy Database 2.0
Metaviridae	Mdg3	Gypsy Database 2.0
Metaviridae	Kabuki	Gypsy Database 2.0
Metaviridae	RetroSor1	Gypsy Database 2.0
Metaviridae	Tat4-1	Gypsy Database 2.0
Metaviridae	Athila4-1	Gypsy Database 2.0
Metaviridae	Diaspora	Gypsy Database 2.0
Metaviridae	Cer4	Gypsy Database 2.0
Metaviridae	SPM	Gypsy Database 2.0
Metaviridae	Mag	Gypsy Database 2.0
Metaviridae	Tor1	Gypsy Database 2.0
Metaviridae	Tor2	Gypsy Database 2.0
Metaviridae	SURL	Gypsy Database 2.0
Metaviridae	Cer3	Gypsy Database 2.0
Metaviridae	Gmr1	Gypsy Database 2.0
Metaviridae	Woot	Gypsy Database 2.0

Metaviridae	Mdg1	Gypsy Database 2.0
Metaviridae	Tor4a	Gypsy Database 2.0
Metaviridae	Tom	Gypsy Database 2.0
Metaviridae	Zam	Gypsy Database 2.0
Metaviridae	Gypsy	Gypsy Database 2.0
Non-LTR	TRAS1	BAA07467.1
Non-LTR	CATS	BAE44464.1
Non-LTR	You	CAC16871.1
Non-LTR	I	AAA70222.2
Non-LTR	Lian-Aa1	AAB65093.1
Non-LTR	CgT1	AAA85636.1
Non-LTR	MGR583	AAB71689.1
Pseudoviridae	1731	Gypsy Database 2.0
Pseudoviridae	pCretro6	Gypsy Database 2.0
Pseudoviridae	SIRE1-4	Gypsy Database 2.0
Pseudoviridae	Vitico1-1	Gypsy Database 2.0
Pseudoviridae	Hydra1-2	Gypsy Database 2.0
Pseudoviridae	Copia	Gypsy Database 2.0
Pseudoviridae	Tnt-1	Gypsy Database 2.0
Pseudoviridae	Koala	Gypsy Database 2.0
Pseudoviridae	Ty1B	Gypsy Database 2.0
Pseudoviridae	Ty4	Gypsy Database 2.0
Pseudoviridae	CoDi6.3	Gypsy Database 2.0
Pseudoviridae	Zeco1	Gypsy Database 2.0
Pseudoviridae	CoDi6.7	Gypsy Database 2.0
Retrovirus	SRV-1	M11841.1
Retrovirus	JSRV	NC_001494.1
Retrovirus	MMTV	NC_001503.1
Retrovirus	LDV	U09568.1
Retrovirus	RSV	NC_001407.1
Retrovirus	HIV-1	NC_001802.1
Retrovirus	FIV	NC_001482.1
Retrovirus	EIAV	NC_001450.1
Retrovirus	HTLV-2	NC_001488.1
Retrovirus	BLV	NC_001414.1
Retrovirus	MuLV	NC_001362.1
Retrovirus	FLV	NC_001940.1
Retrovirus	KoRV	AF151794.2
Retrovirus	REV	NC_006934.1
Retrovirus	WDSV	NC_001867.1
Retrovirus	Xen-1	AJ506107.1
Retrovirus	MuERV-L	Y12713.1
Retrovirus	HERV-L	Ref. 1
Retrovirus	SnRV	NC_001724.1

Retrovirus	BFV	NC_001831.1
Retrovirus	FFV	NC_039242.1
Retrovirus	SFV	EU010385.1
Retrovirus	CoEFV	Ref. 3
Retrovirus	AciFLERV	Ref. 4
Retrovirus	AliFLERV	Ref. 4
Retrovirus	DrFV-2	CAAK05053864.1
Retrovirus	Loki-Bsp	Ref. 2
Retrovirus	Loki-Str	Ref. 2
Retrovirus	Loki-Lch	Ref. 2
Retrovirus	Loki-Lca	Ref. 2
Retrovirus	Loki-Npa	Ref. 2

References

1. Vargiu L, Rodriguez-Tome P, Sperber GO, Cadeddu M, Grandi N, Blikstad V, Tramontano E, Blomberg J. 2016. Classification and characterization of human endogenous retroviruses; mosaic forms are common. Retrovirology 13:7.
2. Wang J, Han GZ. 2021. A Sister Lineage of Sampled Retroviruses Corroborates the Complex Evolution of Retroviruses. Mol Biol Evol 38:1031-1039.
3. Han GZ, Worobey M. 2012. An endogenous foamy-like viral element in the coelacanth genome. PLoS Pathog 8:e1002790.
4. Aiewsakun P, Katzourakis A. 2017. Marine origin of retroviruses in the early Palaeozoic Era. Nat Commun 8:13954.
