# Supplementary material for: A Missing Link between Retrotransposons and Retroviruses
Source: mBio. 2022 Mar 15;13(2):e00187-22. doi: 10.1128/mbio.00187-22 (PMC9040795; doi:10.1128/mbio.00187-22)
Supplement: TABLE S3 [file mbio.00187-22-st003.pdf]

Table S3. Information and location of Odin retrotransposons

Species Name	Odin Name	Location	Length	Domain architecture	Year/Mya
Actinia equina	Odin-Aeq1*	WHPX01000520.1: 30,450-37,610	257	LTR-Spc7 super family-Gag_p30 super family-zf-CCHC-pepsin_retropepsin_like super family-RT_LTR-RT_RNaseH_2[1]-RNase_HI_eukaryote_like-Integrase_H2C2-rve-LTR	1.55
Actinia equina	Odin-Aeq2*	WHPX01000285.1: 113,102-119,462	264	zf-CCHC-RT_like super family-EEP super family-RT_LTR-RT_RNaseH_2-RNase_HI_eukaryote_like-Integrase_H2C2-rve	/
Actinia equina	Odin-Aeq3*	WHPX01000643.1: 288,112-282,245	257	LTR-ZnF_C2HC-pepsin_retropepsin_like super family-RT_LTR-RT_RNaseH_2-RNase_HI_eukaryote_like-Integrase_H2C2-rve-LTR	0
Actinia equina	Odin-Aeq4*	WHPX01000927.1: 145,570-152,730	257	LTR-Gag_p30 super family-zf-CCHC-pepsin_retropepsin_like super family-RT_LTR-RT_RNaseH_2-RNase_HI_eukaryote_like-Integrase_H2C2-rve-LTR	4.66
Anemonia viridis	Odin-Avi1*	OCZR010995229.1	150	RT_LTR-RT_RNaseH_2	/
Anemonia viridis	Odin-Avi2	OCZR011008122.1: 239-6	74	RT_like super family	/
Exaiptasia pallida	Odin-Epa*	NW_018384881.1: 1,565-9,850	257	LTR-Smc super family-COG5222 super family-pepsin_retropepsin_like super family-COG5222 super family-pepsin_retropepsin_like super family-RT_LTR-RT_RNaseH_2-RNase_HI_eukaryote_like-LTR	1.68
Heteractis crispa	Odin-Hc2	JAABNW010172223.1: 4,707,417-4,707,706	89	RT_like super family	/
Heteractis crispa	Odin-Hcr1*	JAABNW010039650.1: 482-1,240	252	RT_LTR	/
Heteractis magnifica	Odin-Hma1*	JAADYU010007112.1: 19,601,242-19,601,656	106	RT_like super family	/
Heteractis magnifica	Odin-Hma2*	JAADYU010014557.1: 2,152-428	139	RT_like super family-RT_RNaseH_2 super family-RT_RNaseH-Integrase_H2C2-rve	/

Heteractis magnifica	Odin-Hma3*	JAADYU010071112.1: 32,032,390-32,031,911	159	RT_LTR	/
Heteractis magnifica	Odin-Hma4*	JAADYU010071112.1: 36,167,908-36,167,528	123	CD_CSD super family-RT_like super family	/
Heteractis magnifica	Odin-Hma5	JAADYU010127380.1: 252-12	87	RT_like super family	/
Heteractis magnifica	Odin-Hma6	JAADYU010200116.1	76	RT_like super family	/
Phymanthus crucifer	Odin-Pcr1*	WUCR01014668.1: 2-1391	162	RT_LTR-RT_RNaseH_2-RNase_H_like super family	/
Phymanthus crucifer	Odin-Pcr2*	WUCR01058184.1: 87,815,408-87,816,527	124	RT_like super family	/
Phymanthus crucifer	Odin-Pcr3*	WUCR01058184.1: 23,215,710-23,216,346	162	RT_LTR-RT_RNaseH_2 super family	/
Phymanthus crucifer	Odin-Pcr4	WUCR01388429.1: 2-211	70	RT_like super family	/
Stichodactyla helianthus	Odin-She1*	WUAP01032288.1: 1-798	161	RT_LTR-RT_RNaseH_2	/
Stichodactyla helianthus	Odin-She2*	WUAP01036653.1: 107-723	166	RT_like super family-RT_RNaseH_2 super family	/
Stichodactyla helianthus	Odin-She3*	WUAP01032092.1: 474-851	121	RT_like super family	/
Stichodactyla helianthus	Odin-She4*	WUAP01075557.1: 5,402,320-5,402,865	136	RT_like super family	/
Stichodactyla mertensii	Odin-Sme1*	JAAAQI010063167.1: 6,439,467-6,439,072	102	RT_LTR	/
Stichodactyla mertensii	Odin-Sme2*	JAAAQI010007685.1: 1,363-1,830	123	RT_like super family	/
Stichodactyla mertensii	Odin-Sme3*	JAAAQI010007527.1: 706-3,060	257	RT_LTR-RT_RNaseH_2-RNase_HI_eukaryote_like-Integrase_H2C2	/
Stichodactyla mertensii	Odin-Sme4	JAAAQI010043222.1: 154,759-155,233	53	RT_like super family-RT_RNaseH_2	/

*sequences used in phylogenetic reconstruction

[1]tether domain
